# Midgut Transcriptional Variation of *Chilo suppressalis* Larvae Induced by Feeding on the Dead-End Trap Plant, *Vetiveria zizanioides*

**DOI:** 10.3389/fphys.2018.01067

**Published:** 2018-08-07

**Authors:** Yanhui Lu, Yanyan Zhao, Han Lu, Qi Bai, Yajun Yang, Xusong Zheng, Zhongxian Lu

**Affiliations:** ^1^State Key Laboratory Breeding Base for Zhejiang Sustainable Pest and Disease Control, Institute of Plant Protection and Microbiology, Zhejiang Academy of Agricultural Sciences, Hangzhou, China; ^2^School of Life Sciences, Lanzhou University, Lanzhou, China

**Keywords:** *Chilo suppressalis*, vetiver, midgut transcriptome, digestion, immunity, detoxification

## Abstract

*Chilo supprressalis* is one of the most important rice pests that causes serious damage to production in the rice growth area of Asia. Vetiver grass (*Vetiveria zizanioides*) was previously found to effectively attract female adults of *C. suppressalis* laying eggs on vetiver leaves, while the larvae cannot complete their life cycles by feeding on vetiver, indicating a potential means of controlling this pest. In the present study, the transcriptomes of midguts of rice-fed and vetiver-fed *C. suppressalis* larvae were profiled, which aimed to clarify the molecular mechanism of vetiver as a dead-end trap plant preliminarily. We found that ingestion of vetiver provoked a robust transcriptional response in the larval midguts, and a total of 1,849 differentially expressed UniGenes were identified. We focused on 12 digestion-related genes, four immune-related genes and three detoxification-related genes. Most of these genes were significantly down regulated in the larval midguts at 6, 8, and 10 days after feeding on vetiver compared to on rice. Transcriptional dynamics suggested that these genes might be involved in toxicity responses following exposure to vetiver. Taken together, this study provides an initial molecular framework for developing biological control strategies for *C. suppressalis* in an effort to protect economically important rice crops.

## Introduction

Rice (*Oryza sativa* L.) is the staple food for half the world’s population. It is frequently attacked by rice stem borers, which are persistent and chronic pests occurring in nearly all paddy fields during each growing season throughout the rice producing area of Asia. Among these stem borers, the striped stem borer *Chilo suppressalis* (Walker) (Lepidoptera: Crambidae) is widely distributed. It is responsible for huge annual yield losses for rice (Qu et al., 2003). *C. suppressalis* has developed resistance to numerous insecticides, which has led to frequent control failures in paddy fields (Lu et al., 2017b). Hence, considerable attention has been paid to trap plants as a means of biological control of *C. suppressalis*.

Vetiver grass (*Vetiveria zizanioides* L.), which is typically planted to stop soil erosion on hills and roads, repels many insect species. Up to now, most studies on vetiver have been carried out with vetiver oil (Zhu et al., 2001; Weyerstahl et al., 2006; Wedler et al., 2016). However, insect infestations of vetiver grass have been reported, and this grass has been identified as a potential trap plant for some crop pests. Our previous results have shown that adult female *C. suppressalis* preferred to lay eggs in vetiver grass, but their larvae cannot survive while feeding on this plant (Zheng et al., 2009; Lu et al., 2017a). Hence, vetiver is a bone fide dead-end trap plant [The term “dead-end trap plant” refers to plants that insects lay eggs on, but their larval offspring cannot survive on the plant as a food source (Shelton and Badenes-Perez, 2006)] for this insect species, and it offers a very promising approach for controlling *C. suppressalis* populations in rice paddies in China (Zheng et al., 2009; Liang et al., 2015; Lu et al., 2015). In fact, vetiver has already been successfully deployed on rice farms to date (Lu et al., 2015), and its nutriological and toxicological mechanisms against the larvae of *C. suppressalis* have been reported recently (Lu et al., 2017a). However, the molecular mechanisms underpinning vetiver’s lethal effects on *C. suppressalis* larvae are currently unclear.

The *C. suppressalis* larval midgut is likely to be important for mediating toxic responses triggered by vetiver ingestion and functions in nutrient uptake and utilization. It plays a vital role in xenobiotic metabolism. Several detoxification enzymes have been identified in the *C. suppressalis* midgut, including carboxylesterase, cytochrome P450 and glutathione *S*-transferase (Huang et al., 2009). Except digestive and detoxification functions, the midgut epithelium serves as a barrier between the internal and external environment. This barrier protects the host against exogenous stresses and maintains intestinal immune homeostasis (Buchon et al., 2009; Bao et al., 2012). In spite of the importance of midgut, little is known about the physiological and immunological responses of this tissue to vetiver in *C. suppressalis*.

Recently, transcriptional profiling has successfully elucidated mechanisms underpinning the insect defense response following exposure to toxic plants (Crava et al., 2016). In the current study, we likewise utilized genome-wide high-throughput RNA sequencing to generate a complete transcriptional profile of the midgut of *C. suppressalis* larvae after ingestion of rice and vetiver. This comparison profiling facilitated the investigation of the complex molecular responses of these larvae to vetiver. Candidate digestion-, detoxification-, and immune-related genes with different expression patterns were selected for additional study and their transcriptional dynamics following vetiver exposure were validated by quantitative real-time PCR (qRT-PCR). Cumulatively, this research provides a transcriptomic resource for further study of the complex responses of the *C. suppressalis* larval midgut after feeding on vetiver, and it offers a rich panel of genes to functionally test for their roles in the toxicity response. This work will be important for the development of effective approaches for sustainable biological control of *C. suppressalis* using vetiver as a trap plant in the economically vital rice production.

## Materials and Methods

### Insects and Plants

*Chilo suppressalis* individuals were collected in 2014 from a rice paddy field in Xiaoshan (120°12′E, 30°04′N), Hangzhou, Zhejiang province. Samples were immediately transferred to the laboratory to maintain genetic diversity in the population. The *C. suppressalis* population was reared *en masse* in an Artificial Atmospheric Phenomena Simulator (Ningbo Jiangnan Instrument Factory, China). Rearing conditions: temperature, 27 ± 1°C; relative humidity (RH), 70-80%; photoperiod of 16 h: 8 h light: dark (L: D). Larvae were reared on an artificial diet (Hu et al., 2012), and adults were fed a 10% honey solution. No specific permissions were required because *C. suppressalis* is not a protected species.

Vetiver grass was propagated from stem cuttings. The vetiver stems were cut from green house and these vetiver grasses were planted in the same time. TN1 rice (*Oryza sativa* L.) was transplanted in pots from 14-day-old seedlings. The TN1 seeds were donated by the International Rice Research Institute, Philippines. All seedlings were grown in a standard potting mix in a climate-controlled room (25 ± 1°C; 16 h: 8 h L: D; 70–80% RH). The stems from 40-day-old plants (vetiver grass and rice) were used for feeding experiments. The health of the vetiver grass and rice seedlings was grown well and not damaged by pests.

### Incubation of *C. suppressalis* Larvae With Vetiver Stems

Third-instar larvae of *C. suppressalis* were selected for the feeding experiments. Experimental larvae were reared on vetiver stems, and control larvae were fed rice stems. All treated larvae were incubated under controlled climate conditions (27 ± 1°C; 70–80% RH; 16 h: 8 h L: D). At 2, 4, 6, 8, and 10 days after feeding, at least 40 larvae were dissected to obtain the midgut for further processing. Both treatments were repeated three times. The samples obtained after 2 days of feeding were used for transcriptional profiling and qRT-PCR validation. All samples were used for qRT-PCR analysis.

### RNA Extraction, cDNA Library Preparation and Illumina Deep-Sequencing Analysis

Total RNA was extracted from 30 *C. suppressalis* midguts using the miRNeasy Mini Kit (Qiagen, United States) according to manufacturer’s protocol. Aimed to check the RNA quality, the absorbance at 260 nm/280 nm (A260/A280) of RNA was measured by a ND-2000 spectrophotometer (ThermoFisher, United States). The purified RNA concentration was determined using a Qubit^®^ 2.0 Fluorometer (Invitrogen, United States). RNA integrity was assessed using the Agilent Technologies 2100 Bioanalyzer (Agilent Technologies, Santa Clara, CA, United States). The 260/280 ratios of all the RNA samples were 1.9–2.0 and the RIN numbers were >8.0. We collected 3.0 μg of total RNA from each sample, which was midgut sample from *C. suppressalis* larvae after 2 days feeding on rice or vetiver, for RNA-Seq library construction and sequencing. After mRNA was purified and fragmented, the NEBNext Ultra RNA Library Prep Kit for Illumina (NEB, United States) was utiltized to establish the cDNA libraires. Six samples were then clustered and sequenced with 2 × 125 bp read lengths on an Illumina Hiseq 2500 platform (Illumina, United States) with TruSeq SBS Kit v4-HS (250-cycles) sequencing kit (Song et al., 2016). All the samples were sequenced on the same lane.

### RNA-Seq Data Analysis

RNA-Seq reads were assessed for quality control with FastQC (version 0.10.1; Babraham Bioinformatics, Cambridge, United Kingdom). All reads were assembled by trinity (trinityrnaseq_r20131110, Grabherr et al., 2011) with default parameters. The transcript abundances were measured as fragments per kilobase of exon per million fragments mapped (FPKM) by RSEM follow trintiy script. Cuffdiff (Trapnell et al., 2013) was then used to determine differential expression (FDR ≤ 0.05) with blind dispersion methods. The raw RNAseq data were submitted to SRA (PRJNA477756).

### Gene Function and Pathway Analysis

The raw data were filtered using Fqfilter -q 20 -p 30 -Q 33 and fqtrimmer_paired -t 30 -Q 33 -l 50 program. The RSeQC (RSeQC-2.3.6) were used to determine the GC content and sequence duplication level. The list of differentially expressed transcripts generated from Cufflinks-Cuffdiff (v2.0.2) was divided into up-regulated and down-regulated lists of transcripts. Gene Ontology and KEGG pathways were determined to be over-represented using the Fisher exact test with a false discovery rate (FDR) correction (FDR ≤ 0.05). 1E-5 setting was used for the BLASTx analyses.

### Validation by qRT-PCR

To validate the transcriptomic data, a subset of differentially expressed genes was quantified by qRT-PCR. The genes were selected based on our transcriptomic data and previous study (Lu et al., 2017a), however, only some related genes were focused on owing to a lot of differential expressed genes identified. Total RNA from each sample was extracted and all RNA samples were treated with DNaseI (ThermoFisher Scientific) following the manufacturer’s protocols. 3.0 μg of total RNA was used to synthesize first-strand cDNA template using a First Strand cDNA Synthesis Kit (ThermoFisher Scientific) with oligo (dT)_18_ as the primer. qRT-PCR on samples prepared with iTaq Universal SYBR Green Supermix (Bio-Rad, Hercules, CA, United States) were performed using the CFX96 Real-Time System (Bio-Rad). The following PCR cycling condition was used: one cycle of 95°C for 30 s; 40 cycles of 95°C for 30 s, and 60°C for 35 s. Melting curve analysis was applied determine the specificity and quantity of the PCR product. Gene-specific primer sequences are listed in **Table 1**. *β-tubulin* (EU429675) served as a reference gene. Relative gene expression values were calculated using the 2^−ΔΔCt^ method (Livak and Schmittgen, 2001). Larval midguts dissected at 2, 4, 6, 8, and 10 days after feeding were also analyzed by qRT-PCR.

**Table 1:** Gene-specific primers used for qRT-PCR.

UniGene number	UniGene name	Sequence [5′-3′]^a^	Product size (bp)^b^
**Digestion-related genes**			
000273	Chymotrypsin-like protein	F: AGGCATGTTCGAAGCTGACA; R: CCACTGGCTGTCAATCTGGT	291
000399	Trypsin-like proteinase	F: CTGTTGGATATACGCCGGCT; R: GGGTGATTGATGATGCGTGC	100
001125	Aminopeptidase N2	F: GCACCAAACGTAACGACACC; R: CCGTTTGCCTGCTCATTTCC	252
005074	Carboxypeptidase A1	F: AGCCGAAACAAAATGGACGC; R: CAGGTCTATCAGGGGTCGGA	202
000365	Carboxypeptidase	F: AGTAATCCTTGCGCCGACAA; R: GTAGAGAACCGTCGTGTCCC	169
000540	Dipeptidyl-peptidase	F: TATCCCAAGGTCGGCACAAC; R: CGACCGGATTTCTTGCACAC	252
001033	Serine protease 20	F: CAGCTGGCACGAATCTGTTG; R: AAACTCCGTCGTACCAGCAG	221
006146	Pancreatic lipase	F: GCCCGGATGTAACCTGAACA; R: AAAGCCCACCCATCCTTAGC	164
000272	Neutral lipase	F: CAGTTTGGGTGCTCACGTTG; R: ATGCGACCGTCTGTGTGAAT	165
000582	Glucosidase	F: CAAGTTTGGCTGGTTCGCTC; R: AGGTGCCCTTGATTCTGTCG	158
000092	Alpha-amylase 1	F: ATCGGCATTCCCTCGATGAC; R: AAACTCCGACGCAGCAGTAA	102
000242	Alpha-amylase 2	F: TGGTGCTGTGGACTTACGAC; R: GCATGTCGGCGAATTGTCTC	264
**Immune response-related genes**			
001314	Peptidoglycan recognition protein	F: GGCTACAACAGTCGCTCCAT; R: AGAGAGCTGACGTCCTCCAT	239
004227	Beta-glucan recognition protein	F: TTAAAGGACGCGTGGCAGAT; R: CTTGGGTGTGATGCGTAGGT	135
005250	Hdd1	F: GTGATCAACGCTGTGCAGATT; R: CTCTCAACACACTCGGGCTG	124
000203	Hdd13	F: GGGGCTGGTGGAGAACTTTT; R: CCACTGAGCTAATACGCCGT	286
**Detoxification-related genes**			
002963	CYP306A1	F: AGCATTTTCGGGGAGAGCTC; R: TCGGGAGATACTGTGCTTGC	154
000438	GSTs-like protein	F: CACCTACCTCGCCAACAAGT; R: CTTTTCTTTATCGGCCGGCG	172
000471	Carboxylesterase	F: GTGCATGCAGATGAAGCTCG; R: TGGCCGTTCTAGAAAGCGTT	192

*^a^F and R refer to forward and reverse primers, respectively. ^b^Length of the amplicon.*

### Statistical Analysis

QRT-PCR data are expressed as the mean ± standard error of triplicate experiments, and statistical significance was determined by *t*-test in the larvae fed on rice and vetiver. Values of *P* < 0.0.5 were considered significant in all treatments.

## Results

### Sequence Analysis and Reads Assembly

**FIGURE 1:**
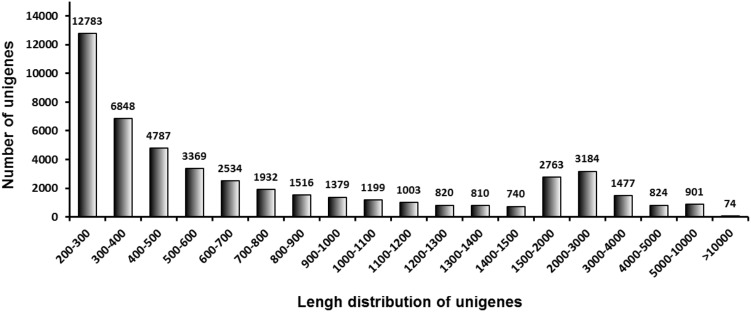
Size distribution of the *C. suppressalis* midgut UniGenes. These include 48,943 UniGenes.

A complete transcriptome was generated by high-throughput RNA sequencing. After removing low-quality and adaptor sequences, 51,810,574 and 53,910,888 clean reads were generated from the midguts of *C. suppressalis* larvae after they were fed for 2 days with rice and vetiver, respectively. The GC-content for each set of reads was about 39.6%, and 76.2–77.0% of the reads mapped to unique sequences. After assembly using the Trinity method, 48,943 UniGenes with an N50 value of 1,874 base pairs (bp) and a mean length of 994.3 bp were obtained (**Table 2**). The largest proportion of UniGenes identified were 200–300 bp in length (26.1%). Size distribution analysis showed that 13,795 UniGenes (28.2%) were more than 1,000 bp, 10,730 UniGenes (21.9%) were 500–1,000 bp and 24,418 UniGenes (49.9%) were 200–500 bp (**Figure 1**).

**Table 2:** Summary of RNA-Seq dataset characteristics.

Reads assembled	Total (nt)
UniGene number	48943
Max UniGene bases	35885
Min UniGene bases	205
Whole dataset lengths	48666252
Average UniGene lengths	994.325
(G+C)/(A+T+G+C)	0.396
N50	1874
N90	375

*N50 is a measure of assembly effect. First, the assembled pieces sort according to fragment lengths and accumulate length values. The accumulated length is greater than or equal to 50% of the total, and the last fragment length is the N50 value. The N90 calculation method is similar to N50.*

### Sequence Homology Distribution

**FIGURE 2:**
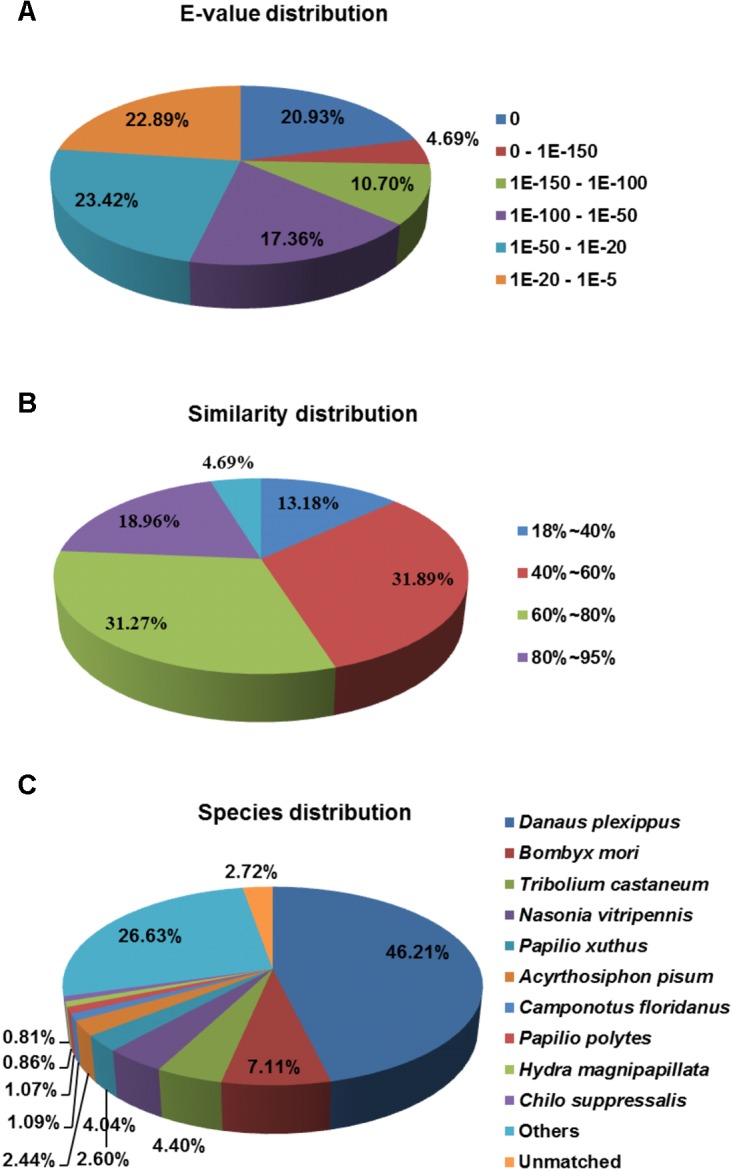
Homology analysis of the *C. suppressalis* midgut UniGenes. All unique gene sequences (20,001) that had BLAST annotations within the NR database with a cut-off *E*-value ≤ 10^–5^ were analyzed for **(A)** *E*-value distribution, **(B)** similarity distribution, and **(C)** species distribution.

To annotate these UniGenes, we searched reference sequences using BLASTx within the non-redundant (NR) NCBI nucleotide database using a cut-off *E*-value of 10^−5^. A total of 28,942 UniGenes (59.1%) did not match any annotated sequences due to both a short nucleotide length and the lack of a sequenced genome for *C. suppressalis*. However, 20,001 UniGenes (40%) displayed annotated BLASTx hits. The *E*-value distributions for the 20,001 annotated UniGenes showed that 36.3% of the sequences had significant homology matches (<1E10^−100^) in the NCBI database, while 63.7% of the sequences ranged from 1E10^−5^ to 1E10^−100^ (**Figure 2A**). The similarity distribution showed that 4.69% of the sequences had greater than 95% homology, 50.23% of the sequences had 60–95% homology, 31.89% of the sequences had 40–60% homology and 13.18% of the sequences had less than 40% homology (**Figure 2B**). A species distribution analysis revealed that 46.21% and 7.11% of the sequences most closely resembled *Danaus plexippus* and *Bombyx mori*, respectively, followed by *Tribolium castaneum* (4.40%) and *Nasonia vitripennis* (4.04%). Approximately 57.0% of the UniGenes were top homology matches for Lepidopterans, including *D. plexippus*, *B. mori*, *Papilio xuthus* and *Papilio polytes*. Only 0.81% of the UniGenes matched *C. suppressalis* sequences previously deposited in the NCBI database (**Figure 2C**).

### Functional Annotation

**FIGURE 3:**
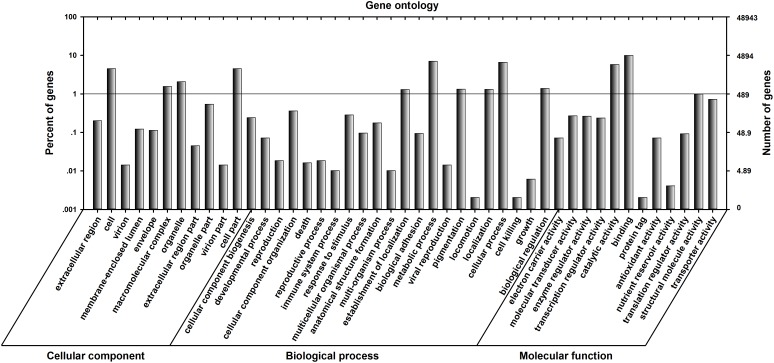
Gene Ontology (GO) classification for the *C. suppressalis* midgut UniGenes. Of the 48,943 UniGenes, 8,239 sequences could be annotated within the GO database into three main categories: cellular component, biological process, and molecular function. The left and right *y*-axes show the percentage and number of genes in each category, respectively.

UniGenes were annotated using the following databases: NCBI non-redundant protein database (NR), Universal Protein (UniProt), Kyoto Encyclopedia of Genes and Genomes (KEGG) and Gene Ontology (GO). A total of 20,818 UniGenes (42.53%) were successfully annotated, including 20,001 (40.87%) from the NR database, 13,980 (28.56%) from the UniProt database, 7,943 (16.23%) from the KEGG database and 8,239 (16.83%) from the GO database. The GO distributions for the UniGenes were classified into three categories (45 subcategories): cellular components (11 subcategories), biological processes (22 subcategories) and molecular function (12 subcategories) (**Figure 3**). For the cellular components category, the clusters relating to “cell part” had an enrichment of 2,222 genes and “cell” had an enrichment of 2,222 genes. For the biological processes category, clusters relating to “metabolic processes” were enriched for 3,450 genes, and those relating to “cellular processes” were enriched for 3,237 genes. For the molecular functions category, clusters relating to “catalytic activity” and “binding” were highly represented (2,849 and 4,867 genes, respectively) (**Figure 3**).

**FIGURE 4:**
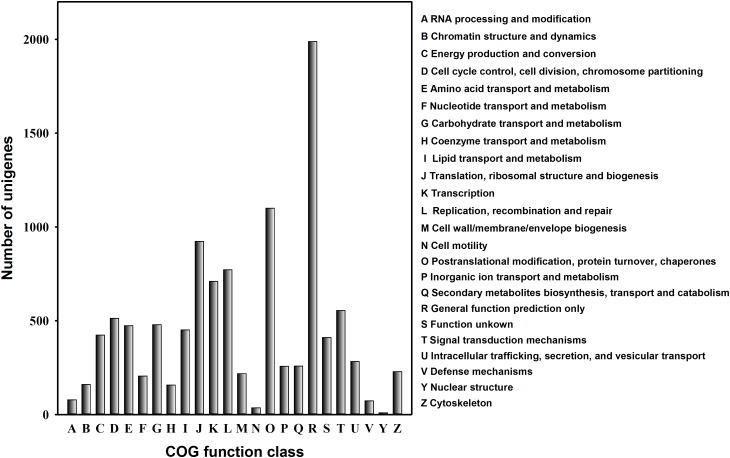
Clusters of Orthologous Groups (COG) classification for the *C. suppressalis* midgut UniGenes. About one-quarter of all UniGenes (10,772) have a COG annotation among the 24 categories.

To further evaluate the effectiveness of annotation, we examined the annotated UniGenes that possessed COG classifications. Of the 20,001 UniGenes, 10,772 sequences had a COG annotation (**Figure 4**). Among the COG categories, the cluster for “General function prediction” represented the largest group (1,989 UniGenes, 18.5%), and followed by “Post-translational modification, protein turnover and chaperones” (1,100 UniGenes, 10.2%) and “Translation, ribosomal structure and biogenesis” (923 UniGenes, 8.6%). The clusters for “RNA processing and modification,” “Defence mechanisms” and “Cell motility” displayed similar abundance (36–79 UniGenes, 0.3–0.7%). In contrast, “Nuclear structure” was the smallest cluster, containing only nine UniGenes (**Figure 4**).

### Complex Response of *C. suppressalis* Midgut to Vetiver Exposure

A total of 1,849 differentially expressed UniGenes were detected in the midguts of *C. suppressalis* larvae that were fed vetiver compared to those that were fed rice. The number of up-regulated genes (1,110) was greater than the number of down-regulated genes (739). In order to study the complex response of the *C. suppressalis* midgut to vetiver exposure, the KEGG database, GO annotations, Genefamily database, and Pfam database were utilized to characterize these differentially expressed UniGenes. Using the KEGG annotations, the differentially expressed UniGenes were enriched for ribosomal, metabolic, and proteasomal pathways (**Supplementary Table S1**). Using GO annotations, the differentially expressed UniGenes were enriched for such categories as translation, ribosomes, intracellular components, structural constituents of the cuticle and chitin binding (**Supplementary Table S2**). Using Genefamily annotations, the differentially expressed UniGenes were enriched for histone folding proteins, DNA/RNA polymerases, serine protease inhibitors and invertebrate chitin-binding proteins (**Supplementary Table S3**). Finally, using Pfam annotations, the differentially expressed UniGenes were enriched for insect cuticle proteins, trypsin inhibitor-like cysteine-rich domain-containing proteins, chitin binding peritrophin-A domain-containing proteins and lipases (**Supplementary Table S4**).

### Quantitative Real-Time PCR (qRT-PCR) Validation

**FIGURE 5:**
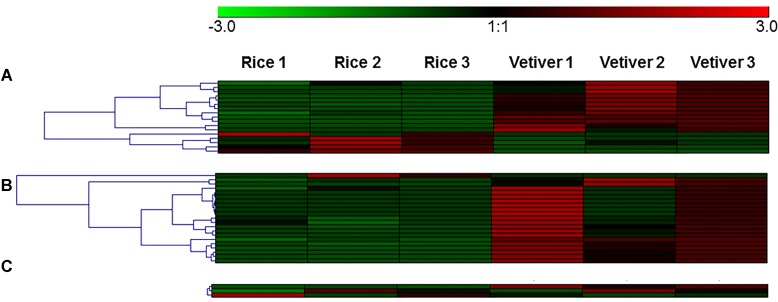
Heatmaps for digestion-related genes **(A)**, immune response-related genes **(B)**, and detoxification-related genes **(C)**.

To validate the transcriptomic data, qRT-PCR was performed for 19 genes that were significantly differentially expressed in the *C. suppressalis* midguts following ingestion of vetiver and rice. These included 12 digestion-related genes, four immune response-related genes and three detoxification-related genes. The results of the qRT-PCR validation are presented as fold-changes normalized to the β*-tublin* gene. A comparison of the mean values for the qRT-PCR results and the original transcriptome data is summarized (**Table 3**). This qRT-PCR analysis validated the results of the transcriptomic analysis. In addition, we generated heatmaps for digestion related genes, immune response-related genes and three detoxification-related genes (**Figure 5**).

**Table 3:** Potential digestion-related, immune response-related, and detoxification-related genes that respond to the ingestion of vetiver grass in the *C. suppressalis* midgut.

UniGene ID	UniGene name	Transcriptome	Real-time PCR	Variation
		Mean ± SE (V/R)^a^	Mean ± SE (V/R)	
**Digestion-related genes**				
000273	Chymotrypsin (*Ostrinia nubilalis*)	3.34 ± 0.44	2.43 ± 0.05	Up
000399	Trypsin (*Chilo suppressalis*)	3.04 ± 0.67	1.85 ± 0.19	Up
001125	Aminopeptidase N2 (*Chilo suppressalis*)	1.15 ± 0.07	3.92 ± 3.11	Up
005074	Carboxypeptidase A1 (*Trichoplusia ni*)	2.54 ± 0.87	1.47 ± 0.85	Up
000365	Carboxypeptidase (*Helicoverpa armigera*)	2.51 ± 0.16	1.45 ± 0.10	Up
000540	Dipeptidyl-peptidase (*Danaus plexippus*)	1.43 ± 0.18	1.54 ± 0.24	Up
001033	Serine protease 20 (*Mamestra configurata*)	4.74 ± 1.04	3.94 ± 0.15	Up
006146	Pancreatic lipase (*Mamestra configurata*)	4.06 ± 1.34	2.82 ± 0.25	Up
000272	Neutral lipase (*Helicoverpa armigera*)	3.95 ± 0.32	2.88 ± 0.17	Up
000582	Glucosidase (*Danaus plexippus*)	1.81 ± 0.40	1.51 ± 0.07	Up
000092	alpha-amylase 1 (*Diatraea saccharalis*)	1.54 ± 0.10	1.15 ± 0.21	Up
000242	alpha-amylase 2 (*Diatraea saccharalis*)	3.54 ± 0.46	2.50 ± 0.47	Up
**Immune response-related genes**				
001314	Peptidoglycan recognition protein (*Papilio xuthus*)	1.75 ± 0.01	2.10 ± 0.17	Up
004227	Glucan recognition protein (*Helicoverpa armigera*)	3.13 ± 1.13	3.92 ± 0.22	Up
005250	Hdd1 (*Danaus plexippus*)	4.82 ± 1.68	1.65 ± 0.21	Up
000203	Hdd13 (*Hyphantria cunea*)	1.73 ± 0.40	1.14 ± 0.56	Up
**Detoxification-related genes**				
002963	CYP306A1 (*Spodoptera littoralis*)	2.37 ± 0.05	1.97 ± 0.28	Up
000438	Glutathione *S*-transferase (*Papilio xuthus*)	1.60 ± 0.16	1.23 ± 0.03	Up
000471	Carboxylesterase protein (*Helicoverpa armigera*)	1.55 ± 0.05	0.38 ± 0.05	–

*^a^V, Vetiver grass; R, Rice; V/R, Gene expression levels in larvae feeding on vetiver grass versus gene expression levels in larvae feeding on rice.*

### Digestion-Related Genes in the *C. suppressalis* Midgut Are Likely Responsive to the Ingestion of Vetiver

**FIGURE 6:**
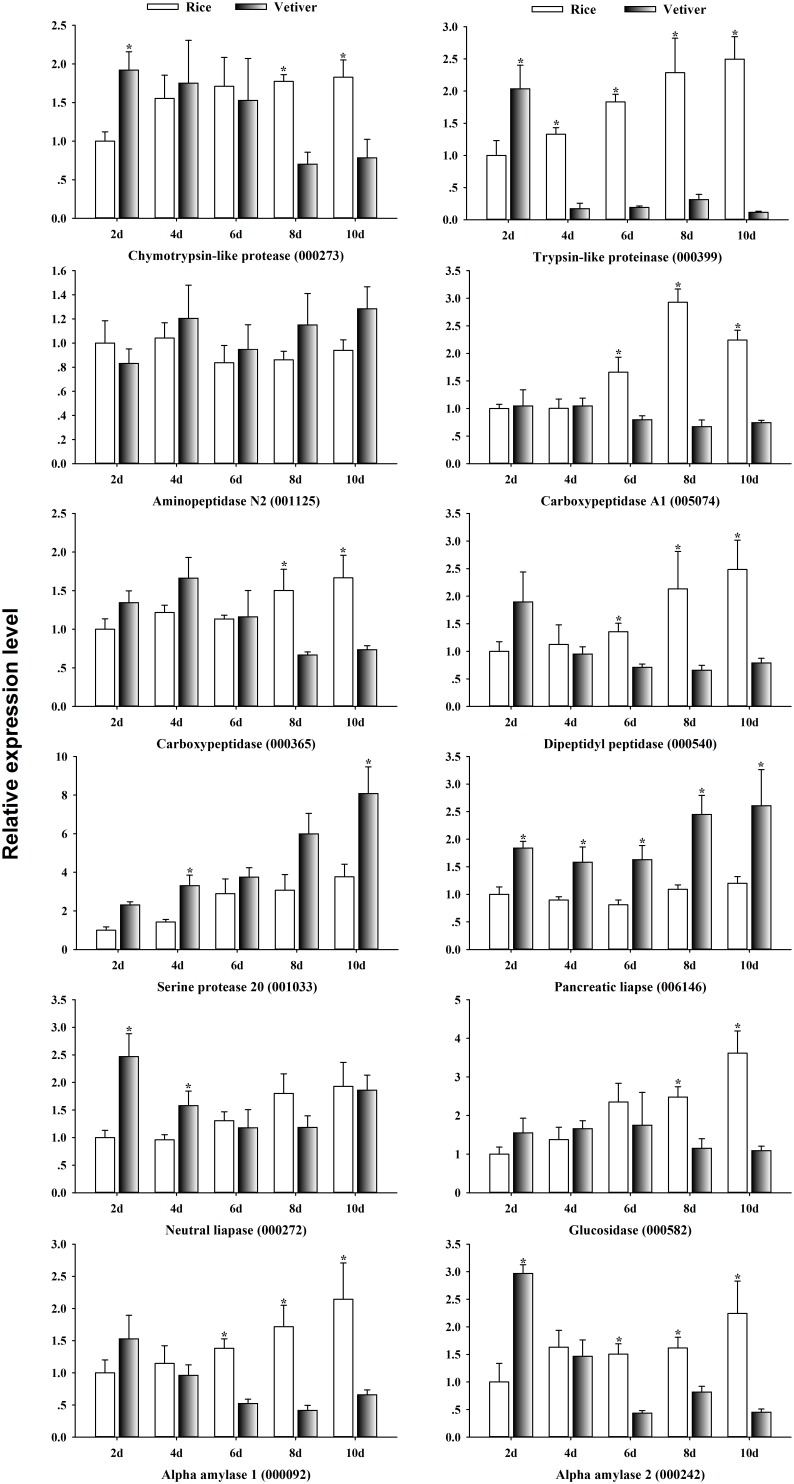
The digestion-related gene expression dynamics in *C. suppressalis* larvae after feeding on either rice or vetiver. Larvae were fed for 2, 4, 6, 8, and 10 days. First-strand cDNA was used as template for each qRT-PCR reaction. The reactions were performed with gene-specific primers to amplify digestion-related genes. The relative expression levels of each gene in each tissue were normalized using *C. suppressalis* β*-tubulin* (EU429675). Threshold cycle (Ct) values were obtained from reactions run on the same plate. Two technical replicates and three biological replicates were analyzed, and the mean ± SEM was calculated to determine the relative transcript levels using the 2^−ΔΔCt^ method. The number in parentheses represents the UniGene IDs. Asterisks indicated significant differences between the gene expressions in the larvae fed on rice and vetiver (*t*-test, *P* < 0.05).

To begin exploring the mechanism of lethality induced by vetiver in larvae of *C. suppressalis*, we randomly selected 12 digestion-related genes from the list of differentially expressed UniGenes and analyzed their expression dynamics in larval midguts at 2, 4, 6, 8, and 10 days after feeding on vetiver or rice (**Figure 6**). The expression levels of three serine proteases, chymotrypsin-like protease (000273) and trypsin-like proteinase (000399) were initially up-regulated and then down-regulated in the larvae feeding on vetiver compared to rice-fed controls. Meanwhile, serine protease 20 (001033) was up-regulated in the larvae feeding on vetiver compared to controls. The expression levels of carboxypeptidase genes (005074, 00365), dipeptidyl peptidase (000540), and glucosidase (000582) were all significantly reduced in larvae at 8 and 10 days after feeding on vetiver compared to the rice-fed controls. Pancreatic lipase (006146) displayed significantly higher expression levels in all larvae that ingested vetiver compared to controls. In contrast, neutral lipase (000272) displayed significantly higher expression levels at 2 and 4 days after feeding on vetiver, but this expression returned to levels comparable to rice-fed control larvae. Two alpha amylases (000092, 000242) showed almost similar expression kinetics in the larvae fed vetiver on different days. However, the expression of aminopeptidase N2 (001125) was not significantly changed in the larvae fed vetiver compared to those fed rice (**Figure 6**).

### Identification of Immune-Related Genes in the *C. suppressalis* Midgut Regulated by the Ingestion of Vetiver

**FIGURE 7:**
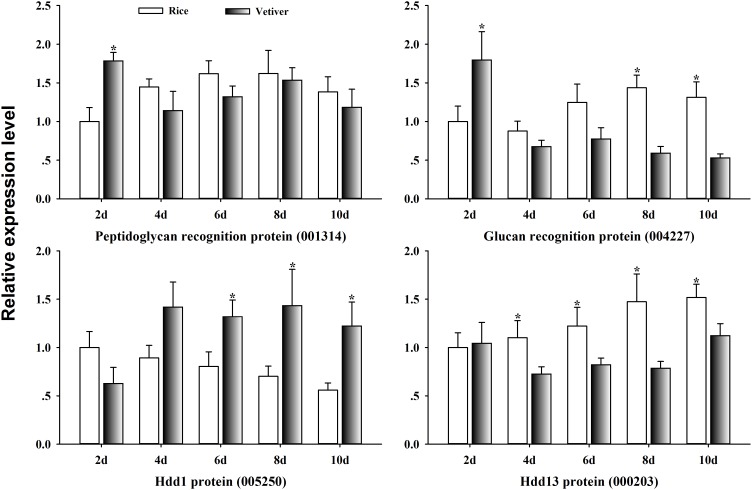
Immune response-related gene expression dynamics in *C. suppressalis* larvae after feeding on either rice or vetiver. Larvae were fed for 2, 4, 6, 8, and 10 days. The qRT-PCR procedure and statistical analysis were performed as described in **Figure 6**. Asterisks indicated significant differences between the gene expressions in the larvae fed on rice and vetiver (*t*-test, *P* < 0.05).

Insects rely on innate immunity for their defense against environmental stresses, including host plants. To clarify the mechanism of lethality of vetiver in the larvae of *C. suppressalis*, we randomly selected four immune-related genes from the list of differentially expressed UniGenes. We assayed their expression kinetics in the larval midgut at 2, 4, 6, 8, and 10 days after feeding on either vetiver or rice using qRT-PCR. Peptidoglycan recognition protein (001314) displayed significantly higher expression in larvae 2 days after feeding on vetiver compared to rice-fed controls; however, this expression subsequently returned to the levels observed in the controls. Glucan recognition protein (004227) exhibited up-regulated and then down-regulated expression kinetics in the larvae feeding on vetiver compared to the controls. Finally, at 4 days after feeding on vetiver, larvae displayed elevated expression of Hdd 1 protein (005250) and reduced expression of Hdd 13 protein (000203) compared to rice-fed controls (**Figure 7**).

### Identification of Detoxification-Related Genes in the *C. suppressalis* Midgut Regulated by the Ingestion of Vetiver

**FIGURE 8:**
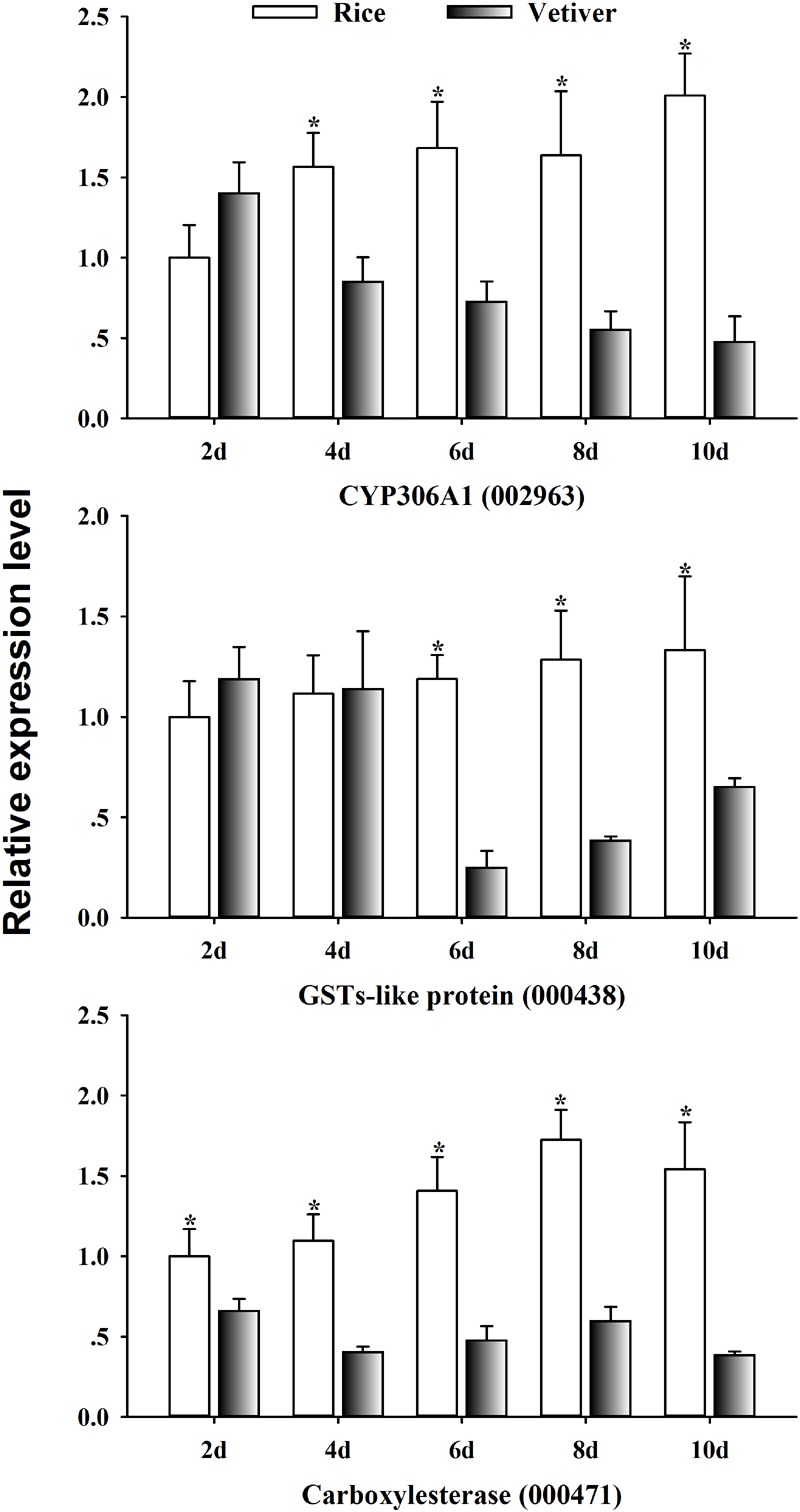
The detoxification-related gene expression dynamics in *C. suppressalis* larvae after feeding on either rice or vetiver. Larvae were fed for 2, 4, 6, 8, and 10 days. The qRT-PCR procedure and statistical analysis were performed as described in **Figure 6**. Asterisks indicated significant differences between the gene expressions in the larvae fed on rice and vetiver (*t*-test, *P* < 0.05).

Cytochrome P450 monooxygenases (P450s), glutathione *S*-transferases (GSTs), and carboxylesterases are members of the three major multigene enzyme families primarily responsible for xenobiotic metabolism (i.e., insecticides and plant-toxic allelochemicals) (Ranson et al., 2002; Bao et al., 2012). To investigate the mechanism of lethality of vetiver in larvae, we randomly selected three detoxification-related genes from the list of differentially expressed UniGenes. We assessed their expression kinetics in the midgut of *C. suppressalis* larvae by qRT-PCR at 2, 4, 6, 8, and 10 days after feeding on vetiver or rice. CYP306A1 (002963) exhibited significantly lower expression levels at 4, 6, 8, and 10 days after feeding on vetiver compared to the rice-fed controls. GST-like protein (000438) displayed significantly lower expression levels at 6, 8, and 10 days after feeding on vetiver compared to controls. Finally, carboxylesterase (000471) displayed significantly higher expression levels in vetiver-fed larvae at all timepoints compared to controls (**Figure 8**).

## Discussion

Recently, transcriptome sequencing has become a crucial research method due to its low cost and high throughput nature (Kang et al., 2013; Song et al., 2016). Here, we report a midgut transcriptomic study of *C. suppressalis.* We identified a large number of high-quality sequences, and investigated the complex responses of the larval midgut after ingestion of vetiver. Due to the lack of a reference genome for *C. suppressalis*, UniGenes were identified and annotated using publicly available protein databases, including the NR, GO, UniProt, KEGG, Swiss-Prot, and Pfam databases. Recently, GO terms have been utilized for the functional annotation and visualization of UniGenes. A framework for categorizing genes has been established, which includes categories consisting of biological process, molecular function, and cellular compartment. In the present study, 8,239 UniGenes were assigned to one or more ontologies. KEGG analysis was utilized for assigning enriched biological pathways to 7,943 UniGenes. Overall, the data provide a useful resource as a *C. suppressalis* transcriptome, yielding insights into the potential genetic cascades that respond to exposure to vetiver.

Previous studies have demonstrated that vetiver effectively attracts female *C. suppressalis* adults, which lay eggs on the plant. However, the larvae cannot complete their life cycle by feeding on vetiver (Zheng et al., 2009). There are two possibly that explain this lethality. First, vetiver may lack certain nutrients present in rice, which leads to digestive dysfunction and, ultimately, larval death. Alternatively, vetiver might contain toxic substances that inhibit the activities of larval detoxification enzymes, leading to fatal metabolic dysfunction (Lu et al., 2017a). Therefore, we explored the potential mechanisms underpinning the observed lethality in *C. suppressalis* larvae. Using qRT-PCR, we investigated the expression dynamics of 12 digestion-related, four immune responsive-related and three detoxification-related UniGenes that were differentially expressed, such as some up-regulated genens, in vetiver-fed larvae compared to rice-fed controls. Digestion is a critical facet of insect physiology (Zibaee et al., 2008), and our results of transcriptome indicated that the expression of numerous digestion-related genes was disrupted in *C. suppressalis* larvae after feeding on vetiver. The expression of most of these selected genes was reduced in larvae at 6 and 8 days after feeding on vetiver, except for aminopeptidase N2 (001125), serine protease 20 (001033), pancreatic lipase (006146) and neutral lipase (000272). This suggested that these digestive enzymes may function in the breakdown of vetiver in the larval midgut, and they may play important roles in absorption and utilization of nutrients from this plant. To date, most studies have focused on the effects that host plants have on the digestive enzymes on insects that feed on them. In particular, host plants have been shown to have significant effects on the activities of insect amylase, pectinase and pepsin (Yan et al., 2012). The amylase and lipase activities of *Spodoptera exigua*, for example, depend on the host plant, which includes maize, sugar beets and cucumber (Zhang et al., 2009). Furthermore, when the action of these digestive enzymes is inhibited, the ability of the insect to derive nutrition from the plant becomes impaired, and larval growth and development are retarded. This eventually leads to starvation and death (Zibaee et al., 2008). Therefore, we suggest that the lethality induced by vetiver in *C. suppressalis* larvae may be related to the inhibition of digestive enzymes. However, a detailed molecular mechanism must be elucidated in order to determine whether impaired digestive enzymes cause lethality in vetiver-fed larvae.

Insects rely on innate immunity for their defense against environmental stresses. Peptidoglycans, beta-1,3-glucan and lipopolysaccharides frequently elicit immune responses in insects (Bao et al., 2012). Accordingly, a variety of pattern recognition proteins mediate immune responses and induce the expression of immune-responsive genes (Cerenius et al., 2010; Bao et al., 2012). However, even though *C. suppressalis* is one of the most serious agricultural pests, little is known about its immune responses to vetiver. This is critical for identifying potential target molecules in this insect’s immune system. Beta-glucan recognition protein is a particularly important pattern recognition molecule in the insect immune system (Bao et al., 2012). In this study, we found that the expression level of beta-glucan recognition protein (004227) significantly decreased 8 days after the larvae were fed vetiver compared to the controls. This suggests that beta-glucan recognition protein might be inhibited in the midgut by the presence of vetiver. Furthermore, among the immune response-related UniGenes, *Hdd1*, and *Hdd13* showed opposing expression dynamics. *Hdd1* (005250) displayed very high transcript levels in larvae 6 days after feeding on vetiver, while *Hdd13* (000203) displayed low transcript levels in larvae 4 days after feeding on vetiver. This suggested that these two genes might have different functions in *C. suppressalis* larvae during the digestion of vetiver.

Most insect species metabolize xenobiotics, such as secondary plant chemicals and insecticides, using a suite of detoxification enzymes. These include P450s, GSTs and carboxylesterases (Karatolos et al., 2011; Bao et al., 2012). In this study, CYP306A1 (002963), GST-like protein (000438), and carboxylesterase (000471) exhibited significantly reduced expression in larvae 6, 8, and 10 days after feeding on vetiver. This finding supported our previous results, suggesting that vetiver contains toxic substances that have lethal effects on *C. suppressalis* larvae. These substances likely inhibited the activities of detoxification enzymes, leading to disruptions in larval metabolism and toxicity responses (Lu et al., 2017a).

## Conclusion

In this study, a transcriptome for *C. suppressalis* has been generated, and the overall midgut responses of the larvae ingestion of vetiver have been described for the first time. We validated this transcriptome by examining the expression of 12 digestion-related, four immune response-related, and three detoxification-related UniGenes by qRT-PCR in larvae that were fed on vetiver. Our results suggest that these genes are possibly involved in the mechanism of lethality induced by trap plant-vetiver in *C. suppressalis* larvae. These transcriptomic data establish a foundation for the identification and functional validation of differentially expressed genes of *C. suppressalis* after vetiver exposure. Overall, our findings provide a molecular framework for the development of a sustainable control approach for *C. suppressalis*, which has potentially beneficial implications for the rice production worldwide.

## Author Contributions

YL, YZ, HL, and ZL conceived and designed the experiments and analyzed the data. YL, YZ, HL, QB, and YY performed the experiments. YL, XZ, and ZL contributed reagents and materials. YL and ZL wrote the manuscript.

## Conflict of Interest Statement

The authors declare that the research was conducted in the absence of any commercial or financial relationships that could be construed as a potential conflict of interest.
