# Supplementary material for: Midgut Transcriptional Variation of Chilo suppressalis Larvae Induced by Feeding on the Dead-End Trap Plant, Vetiveria zizanioides
Source: Front Physiol. 2018 Aug 7;9:1067. doi: 10.3389/fphys.2018.01067 (PMC6090068; doi:10.3389/fphys.2018.01067)
Supplement: Supplementary file 1 [file Table_1.DOC]

**Midgut transcriptional variation of *Chilo suppressalis* larvae induced by feeding on the dead-end trap plant, *Vetiveria zizanioides***

Yanhui Lu^1^, Yanyan Zhao^1^, Han Lu^2^, Qi Bai^1^, Yajun Yang^1^, Xusong Zheng^1^, Zhongxian Lu^1*^

^1^ State Key Laboratory Breeding Base for Zhejiang Sustainable Pest and Disease Control, Institute of Plant Protection and Microbiology, Zhejiang Academy of Agricultural Sciences, Hangzhou 310021, China

^2^ School of Life Sciences, Lanzhou University, Lanzhou 730000, China

^*^Corresponding author: Zhongxian Lu

Tel: +86 571 86404077

Fax: +86 571 86400481

Postal address: No 198, Shiqiao Road, Hangzhou 310021, China

Email: [luzxmh@163.com](mailto:luzxmh@163.com)

**Table S1. Number of differentially expressed genes identified by KEGG analysis (FDR < 0.05).**

| **Annotation ID** | **Function** | **Gene number** | ***P*-value** | **FDR** |
| --- | --- | --- | --- | --- |
| KO 03010 | Ribosome | 112 | 0 | 0 |
| KO 01100 | Metabolic pathways | 88 | 3.10×10^-11^ | 6.52×10^-9^ |
| KO 05016 | Huntington’s disease | 30 | 6.30×10^-11^ | 1.32×10^-8^ |
| KO 05322 | Systemic lupus erythematosus | 15 | 1.58×10^-10^ | 3.32×10^-8^ |
| KO 05012 | Parkinson’s disease | 20 | 8.13×10^-9^ | 1.71×10^-6^ |
| KO 00190 | Oxidative phosphorylation | 20 | 9.27×10^-8^ | 1.95×10^-6^ |
| KO 03050 | Proteasome | 14 | 3.53×10^-6^ | 7.41×10^-4^ |
| KO 05010 | Alzheimer’s disease | 20 | 4.40×10^-6^ | 9.25×10^-4^ |
| KO 00051 | Fructose and mannose metabolism | 9 | 1.48×10^-5^ | 3.10×10^-3^ |
| KO 00240 | Pyrimidine metabolism | 15 | 2.01×10^-5^ | 4.21×10^-3^ |
| KO 03040 | Spliceosome | 16 | 5.00×10^-5^ | 1.05×10^-2^ |
| KO 01110 | Biosynthesis of secondary metabolites | 27 | 7.37×10^-5^ | 1.55×10^-2^ |
| KO 00052 | Galactose metabolism | 9 | 8.89×10^-5^ | 1.87×10^-2^ |
| KO 00100 | Steroid biosynthesis | 6 | 1.59×10^-4^ | 3.32×10^-2^ |
| KO 00230 | Purine metabolism | 16 | 1.90×10^-4^ | 3.99×10^-2^ |
| KO 00620 | Pyruvate metabolism | 9 | 1.99×10^-4^ | 4.18×10^-2^ |

**Table S2. Number of differentially expressed genes identified by GO analysis (FDR < 0.05).**

| **Annotation ID** | **Function** | **Gene number** | ***P*-value** | **FDR** |
| --- | --- | --- | --- | --- |
| GO: 0006412 | Translation | 96 | 0 | 0 |
| GO: 0005840 | Ribosome | 96 | 0 | 0 |
| GO: 0005622 | Intracellular | 82 | 0 | 0 |
| GO: 0042302 | Structural constituent of cuticle | 34 | 0 | 0 |
| GO: 0003735 | Structural constituent of ribosome | 97 | 0 | 0 |
| GO: 0046982 | Protein heterodimerization activity | 22 | 2.89×10^-15^ | 6.06×10^-13^ |
| GO: 0005576 | Extracellular region | 20 | 1.78×10^-12^ | 3.74×10^-10^ |
| GO: 0008061 | Chitin binding | 16 | 5.28×10^-12^ | 1.11×10^-9^ |
| GO: 0006030 | Chitin metabolic process | 15 | 3.26×10^-12^ | 6.85×10^-9^ |
| GO: 0003676 | Nucleic acid binding | 71 | 6.65×10^-11^ | 1.40×10^-8^ |
| GO: 0015074 | DNA integration | 30 | 2.58×10^-9^ | 5.41×10^-7^ |
| GO: 0006351 | Transcription, DNA-templated | 13 | 2.41×10^-8^ | 5.06×10^-6^ |
| GO: 0015979 | Photosynthesis | 7 | 4.00×10^-8^ | 8.40×10^-6^ |
| GO: 0003968 | RNA-directed 5’-3’ RNA polymerase activity | 6 | 7.27×10^-8^ | 1.53×10^-5^ |
| GO: 0009055 | Electron carrier activity | 9 | 3.28×10^-7^ | 6.89×10^-5^ |
| GO: 0043565 | Sequence-specific DNA binding | 15 | 4.46×10^-7^ | 9.36×10^-5^ |
| GO: 0003723 | RNA binding | 19 | 1.12×10^-6^ | 2.36×10^-4^ |
| GO: 0006508 | Proteolysis | 27 | 2.97×10^-6^ | 6.23×10^-4^ |
| GO: 0006629 | Lipid metabolic process | 10 | 7.99×10^-6^ | 1.68×10^-3^ |
| GO: 0003677 | DNA binding | 34 | 1.34×10^-5^ | 2.81×10^-3^ |
| GO: 0003824 | Catalytic activity | 37 | 1.61×10^-5^ | 3.38×10^-3^ |
| GO: 0019843 | rRNA binding | 5 | 1.66×10^-5^ | 3.49×10^-3^ |
| GO: 0005525 | GTP binding | 18 | 1.68×10^-4^ | 3.53×10^-2^ |
| GO: 0003700 | Transcription factor activity, sequence-specific DNA binding | 12 | 1.82×10^-4^ | 3.82×10^-2^ |

**Table S3. Number of differentially expressed genes identified by Genefamily analysis (FDR<0.05).**

| **Annotation ID** | **Superfamily** | **Gene number** | ***P*-value** | **FDR** |
| --- | --- | --- | --- | --- |
| SSF47113 | Histone-fold | 22 | 1.11×10^-15^ | 2.33×10^-13^ |
| SSF53098; SSF56672 | Ribonuclease H-like; DNA/RNA polymerases | 19 | 9.67×10^-12^ | 2.03×10^-9^ |
| SSF57567 | Serine protease inhibitors | 8 | 1.38×10^-9^ | 2.90×10^-7^ |
| SSF57829 | Zn-binding ribosomal proteins | 9 | 2.46×10^-9^ | 5.16×10^-7^ |
| SSF55315 | L30e-like | 9 | 6.95×10^-9^ | 1.46×10^-7^ |
| SSF46946 | S13-like H2TH domain | 5 | 1.95×10^-8^ | 4.10×10^-6^ |
| SSF53474 | Alpha/beta-Hydrolases | 23 | 2.18×10^-7^ | 4.58×10^-5^ |
| SSF56436 | C-type lectin-like | 8 | 7.72×10^-7^ | 1.62×10^-4^ |
| SSF57625 | Invertebrate chitin-binding proteins | 9 | 1.34×10^-6^ | 2.81×10^-4^ |
| SSF54189 | Ribosomal proteins S24e, L23 and L15e | 6 | 2.39×10^-6^ | 5.01×10^-4^ |
| SSF103511 | Chlorophyll a-b binding protein | 5 | 1.07×10^-6^ | 2.25×10^-3^ |
| SSF51430 | NAD(P)-linked oxidoreductase | 6 | 1.12×10^-5^ | 2.35×10^-3^ |
| SSF53137 | Translational machinery components | 5 | 2.49×10^-5^ | 5.22×10^-3^ |
| SSF50182 | Sm-like ribonucleoproteins | 6 | 2.86×10^-5^ | 6.00×10^-3^ |
| SSF56219; SSF56672 | DNase I-like; DNA/RNA polymerases | 10 | 3.40×10^-5^ | 7.15×10^-3^ |
| SSF54495 | UBC-like | 8 | 1.50×10^-4^ | 3.16×10^-2^ |

**Table S4. Number of differentially expressed genes identified by Pfam analysis (FDR < 0.05).**

| **Annotation ID** | **Function** | **Gene number** | ***P*-value** | **FDR** |
| --- | --- | --- | --- | --- |
| PF00379 | Insect cuticle protein | 34 | 0 | 0 |
| PF01826 | Trypsin inhibitor like cysteine rich domain | 10 | 1.24×10^-11^ | 2.60×10^-9^ |
| PF01607 | Chitin binding Peritrophin-A domain | 14 | 1.02×10^-10^ | 2.14×10^-8^ |
| PF00125 | Core histone H2A/H2B/H3/H4 | 13 | 2.22×10^-10^ | 4.66×10^-8^ |
| PF01248 | Ribosomal protein L7Ae/L30e/S12e/Gadd45 family | 10 | 5.90×10^-10^ | 1.24×10^-7^ |
| PF00665 | Integrase core domain | 27 | 1.39×10^-8^ | 2.92×10^-6^ |
| PF00416 | Ribosomal protein S13/S18 | 5 | 1.95×10^-8^ | 4.10×10^-6^ |
| PF05380 | Pao retrotransposon peptidase | 16 | 9.22×10^-8^ | 1.94×10^-5^ |
| PF01391 | Collagen triple helix repeat | 6 | 1.52×10^-7^ | 3.20×10^-5^ |
| PF06585 | Haemolymph juvenile hormone binding protein | 9 | 2.40×10^-7^ | 5.04×10^-5^ |
| PF00151 | Lipase | 9 | 1.03×10^-6^ | 2.16×10^-4^ |
| PF03564 | Protein of unknown function | 12 | 1.92×10^-6^ | 4.03×10^-4^ |
| PF04083 | Partial alpha/beta-hydrolase lipase region | 5 | 3.93×10^-6^ | 8.25×10^-4^ |
| PF07898 | Protein of unknown function | 5 | 6.66×10^-6^ | 1.40×10^-3^ |
| PF01423 | LSM domain | 6 | 7.89×10^-6^ | 1.66×10^-3^ |
| PF00504 | Chlorophyll A-B binding protein | 5 | 1.07×10^-5^ | 2.25×10^-3^ |
| PF00808 | Histone-like transcription factor and archaeal histone | 5 | 1.07×10^-5^ | 2.25×10^-3^ |
| PF00248 | Aldo/keto reductase family | 6 | 1.56×10^-5^ | 3.27×10^-3^ |
| PF00561 | Alpha/beta hydrolase fold | 5 | 5.11×10^-5^ | 1.07×10^-2^ |
| PF00059 | Lectin C-type domain | 6 | 8.12×10^-5^ | 1.71×10^-2^ |
| PF00089 | Trypsin | 13 | 1.13×10^-4^ | 2.37×10^-2^ |
| PF14227 | Gag-polypeptide of LTR copia-type | 6 | 1.59×10^-4^ | 3.33×10^-2^ |
